# Glycans as Modulators of Plant Defense Against Filamentous Pathogens

**DOI:** 10.3389/fpls.2018.00928

**Published:** 2018-07-04

**Authors:** Chayanika Chaliha, Michael D. Rugen, Robert A. Field, Eeshan Kalita

**Affiliations:** ^1^Department of Molecular Biology and Biotechnology, Tezpur University, Tezpur, India; ^2^Department of Biological Chemistry, John Innes Centre, Norwich Research Park, Norwich, United Kingdom

**Keywords:** carbohydrates, glycans, elicitors, priming, plant defense, fungi, oomycete

## Abstract

Plants and microbes utilize glycoconjugates as structural entities, energy reserves for cellular processes, and components of cellular recognition or binding events. The structural heterogeneity of carbohydrates in such systems is a result of the ability of the carbohydrate biosynthetic enzymes to reorient sugar monomers in a variety of forms, generating highly complex, linear, branched, or hierarchical structures. During the interaction between plants and their microbial pathogens, the microbial cell surface glycans, cell wall derived glycans, and glycoproteins stimulate the signaling cascades of plant immune responses, through a series of specific or broad spectrum recognition events. The microbial glycan-induced plant immune responses and the downstream modifications observed in host-plant glycan structures that combat the microbial attack have garnered immense interest among scientists in recent times. This has been enabled by technological advancements in the field of glycobiology, making it possible to study the ongoing co-evolution of the microbial and the corresponding host glycan structures, in greater detail. The new glycan analogs emerging in this evolutionary arms race brings about a fresh perspective to our understanding of plant–pathogen interactions. This review discusses the role of diverse classes of glycans and their derivatives including simple sugars, oligosaccharides, glycoproteins, and glycolipids in relation to the activation of classical Pattern-Triggered Immunity (PTI) and Effector-Triggered Immunity (ETI) defense responses in plants. While primarily encompassing the biological roles of glycans in modulating plant defense responses, this review categorizes glycans based on their structure, thereby enabling parallels to be drawn to other areas of glycobiology. Further, we examine how these molecules are currently being used to develop new bio-active molecules, potent as priming agents to stimulate plant defense response and as templates for designing environmentally friendly foliar sprays for plant protection.

## Introduction

Plant cell walls are complex configurations of highly recalcitrant interlocking polysaccharides which insulate against microbial invasion and abiotic stress. Filamentous plant pathogens, which mainly comprise fungi and oomycetes, breach the plant cell wall by releasing enzymes that deconstruct polysaccharides, proteins, and lignin based polymers (Zhao et al., 2013). The resulting breakdown products that accumulate in apoplastic fluids represent the first molecular interaction between microbes and the plant. As such, the apoplast also represents the zone where the plants need to distinguish between symbionts and pathogens, based on their molecular signatures (Okmen and Doehlemann, 2016).

**FIGURE 1:**
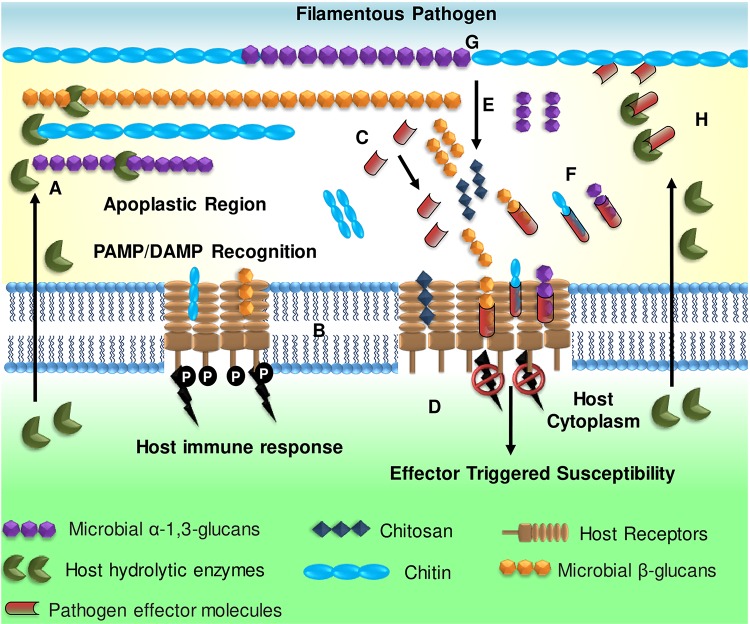
Overview of glycan-triggered immune responses during plant–filamentous pathogen interactions. **(A)** Filamentous pathogen cell walls are targeted by host-derived hydrolytic enzymes, as an innate defense response resulting in the release of glycan fragments (chitin, α- and β-glucans). **(B)** The hydrolyzed glycans are recognized as PAMPs by host pattern recognition receptors (PRRs) triggering innate immune response. **(C)** The pathogen secretes effector molecules that **(D)** suppress PTI defense responses by **(E)** converting immunogenic chitin to the less immunogenic chitosan **(F)** sequestering or masking the PAMPs released, to evade detection. **(G)** Remodeling of the cell wall components by the pathogen (e.g., accumulation of α-1,3-glucan) mitigates the effect of host-hydrolytic enzymes on the microbial cell wall glycans, thereby preventing hydrolysis. **(H)** Lastly, the pathogens may secrete certain effectors that directly inhibit host hydrolytic enzymes. (Adapted from Rovenich et al., 2016 and reproduced with permission from John Wiley and Sons).

**FIGURE 2:**
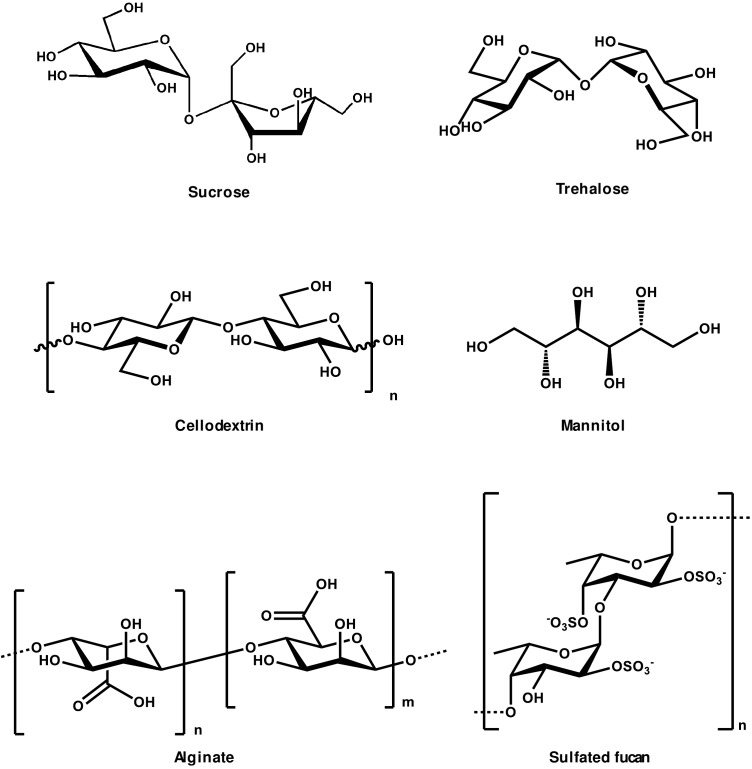
Chemical structures of oligosaccharides showing elicitor activity.

The activation of the complex array of plant innate defense mechanisms relies on the recognition of pathogen signatures by the host transmembrane pattern recognition receptors (PRRs) (Zipfel, 2014). The signatures perceived by PRRs are conserved molecular patterns called PAMPs/MAMPs (Pathogen or Microbe Associated Molecular Patterns) which are either the breakdown products resulting from microbial enzyme action or pathogenic effectors secreted by microbes to promote infection (**Figures 1, 2** and **Table 1**; Trouvelot et al., 2014). However, pathogens also secrete effectors to mask the PTI response resulting from PAMP recognition by the host. The secretion of these effectors mediate the remodeling of the microbial cell wall due to which the pathogen is able to either escape the host defense or trigger the ETI responses (Hopke et al., 2018). This has been seen in mammalian systems, yet its importance in carbohydrate mediated interactions between plants and microbes remains to be determined.

**Table 1:** Various carbohydrate elicitors inducing defense responses in plants.

Carbohydrate elicitors	Source	Plants eliciting defense responses	Plant defense responses	Reference
Sugars (Glucose, G-6-P,Sucrose, Trehalose, T-6-P)	Plant	*Arabidopsis, Lupinus angustifolius*, wheat	Activation of pathogen resistance (PR) genes, phenylalanine ammonia-lyase (PAL), and peroxidase (POX) activity	Xiao et al., 2000; Reignault et al., 2001; Zhang et al., 2009; Nunes et al., 2013
β-glucans	Microbial cell wall	*Arabidopsis*, leguminous plant, grapevine, alfalfa, tobacco	Induction of chitinase and PAL activity, synthesis of isoflavanoid and phytoalexins	Klarzynski et al., 2000; Caillot et al., 2012; Fesel and Zuccaro, 2016
Laminarin	Brown algae	Tobacco, grapevine	Induction of calcium influx, oxidative burst, activation of PR genes	Klarzynski et al., 2000; Aziz et al., 2003
Chitin	Fungal cell wall	Rice, barley, *Arabidopsis,*	Induce hypersensitive response, mitogen activated protein kinase (MAPK) pathway, PR genes, chitinase activity, PAL activity, phytoalexins accumulation, callose deposition	Yamada et al., 1993; Kishimoto et al., 2010; Tanaka et al., 2010; Yamaguchi et al., 2013,
Chitosan	Fungal cell wall	Pea, dicotyledonous species, melon	Induce PR genes, chitinase activity, PAL activity, phytoalexin accumulation	Kohle et al., 1985; Conrath et al., 1989; Burkhanova et al., 2007
Cellodextrins	Plant	Grapevine	Activation of PR genes, induction of calcium influx, oxidative burst, phytoalexin accumulation	De Lorenzo and Ferrari, 2002; Aziz et al., 2007
Alginate	Sea weed	Soybean, rice	Phytoalexins accumulation, activation of PR genes encoding PAL, induction of POX activity	An et al., 2009 ; Zhang S. et al., 2015
Fucans	Sea weed	Tobacco	Induce PAL activity, PR genes	Kombrink and Somssich, 1995; Klarzynski et al., 2003


Although PAMPs represent a broad range of molecules, which includes carbohydrates, lipids, proteins, peptides, lipopolysaccharides, glycolipids, and glycoproteins (Boller and Felix, 2009; Trouvelot et al., 2014), it is only during recent times that advances in functional glycomics have encouraged researchers to analyze the role of glycans in plant–pathogen interactions. The threat to food security from filamentous pathogens, which rely on a host of glycans to mediate pathogenesis, accounting for an estimated loss of 10–40% of total crop production worldwide (Sobhy et al., 2014; Anderson et al., 2016) has driven intense research in this area. In turn, this had led to concepts like “sweet immunity” and “sugar enhanced defense” that explore the multi-faceted and systemic role of carbohydrates as modulators of plant immunity (Trouvelot et al., 2014; Rovenich et al., 2016).

In the context of glycan-mediated plant immunity, identification of microbial cell-surface glycans, building blocks of fungal/oomycetes cell walls (e.g., chitin and β-glucans) and bacterial glycoconjugates (e.g., lipopolysaccharide, glycoproteins, and lectins) that act as regulators of plant defense signaling, presents new perspectives to analyze and understand host–microbe interactions (Vidal et al., 1998; Boudart et al., 2003; Trouvelot et al., 2014). Within this review, we look at how the different classes of glycans become a part of the strategic interactions during plant–microbe interaction and their future potential as defense priming agents for plant protection. The review covers a broad range of topics, providing a brief insight that can act as a primer to an audience unfamiliar with this topic alongside those studying plant–pathogen interaction.

## Simple Sugars: Mono and Di-Saccharides

Simple sugars including mono and di-saccharides are central to plant–microbe interactions, serving both as energy sources to drive the PTI and ETI responses as well as themselves acting as signaling molecules that drive signal fluxes leading to localized or systemic defense responses, when challenged with filamentous pathogens (Trouvelot et al., 2014).

### Glucose, Sucrose, and Associated Metabolites

The role of the glucose sensor Hexokinase (HXK), which is responsible for the conversion of glucose to glucose 6-phosphate, has been most investigated. Among the several isoforms of HXK1, the mitochondria-associated HXK1 is central to the control of programmed cell death (PCD) during microbial pathogenesis and is also responsible for regulation of several pathogenesis related (*PR*) genes, during HR mediated cell death (Kim et al., 2006). Glucose also activates the expression of several PR genes, of which some may require the HXK to be catalytically active. For instance, glucose mediated the induction of *PR-1* and *PR-5* in *Arabidopsis* in an *AtHXK1* dependent manner and also *Arabidopsis* lines overexpressing mitochondrial HXK have higher basal transcript levels of *PR* genes showing enhanced resistance to the necrotrophic fungal pathogen *Alternaria brassicicola* (Xiao et al., 2000; Rojas et al., 2014).

Sucrose has emerged as an important molecule in plant sugar signaling networks owing to recent evidences of its involvement in the modulation of innate immunity and defense responses during microbial attack (Gomez-Ariza et al., 2007; Bolouri Moghaddam and Van den Ende, 2012). It is suggested that cell-wall localized invertases hydrolyze sucrose to generate glucose, which in turn act as signal fluxes that are sensed by HXKs to activate downstream defense signaling (Moore et al., 2003; Cho et al., 2009; Tauzin and Giardina, 2014). Additionally, sucrose has been seen to drive the expression of secondary metabolite synthesis pathways including anthocyanin and isoflavonoid production, as a defense response against *Fusarium oxysporum* in *Lupinus angustifolius* and in embryo axes of *Lupinus luteus* L. cv. Juno (Morkunas et al., 2005; Formela et al., 2014).

**FIGURE 3:**
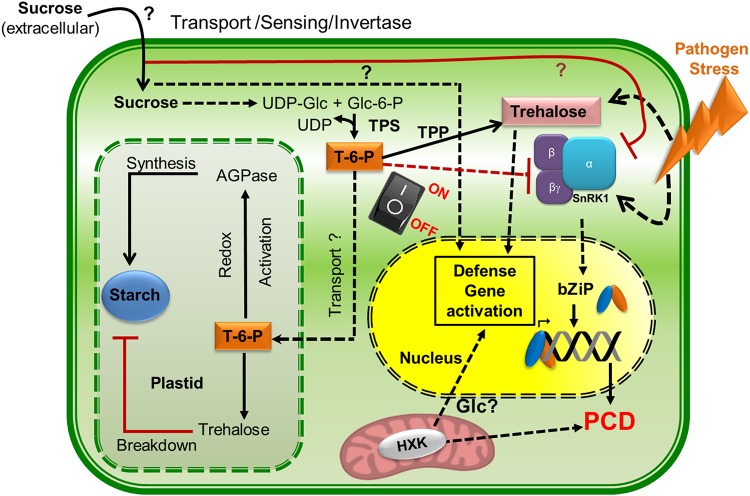
Sugar fluxes regulating defense responses under pathogen attack. Trehalose-6-phosphate (T-6-P) is central to the metabolic switch between energy intensive processes like starch synthesis and low sugar stress conditions (when under pathogen attack or during plant defense signaling). T-6-P regulates the switch between these two conditions by regulating the multi-functional heterotrimeric stress regulator, SnRK1. SnRK1 can perceive low sugar stress and mediate defense against pathogens through post translational modification of key metabolic enzymes, activating PCD and bZIP (basic leucine zipper) mediated transcriptional reprogramming. Trehalose on the other hand can also sense pathogen stress and respond by activating defense genes and blocking the energy intensive starch biosynthesis. Sucrose regulates defense signaling both positively and negatively by activating secondary metabolite production under low sugar conditions on one hand and inhibiting SnRK1 on the other, during normal conditions. Additionally, the mitochondrial HXKs are also implicated in activating PCD and defense gene activation during pathogen attack. However, little is known if Glucose mediates the process. Thus, the components of signalling pathway of sugars (like sucrose, glucose and trehalose) that maintains the balance between stress conditions and homeostatsis are yet to be discovered and are indicated by “?” in the figure.

Trehalose and trehalose-6-phosphate (T-6-P) are considered important sugar signals, modulating defense responses through complex sugar sensing pathways (**Figure 3**). Trehalose induces the activation of the defense genes Phenylalanine Ammonia-Lyase (PAL) and Peroxidase (POX) during wheat challenge with *Blumeria graminis*, the causal agent of powdery mildew disease (Reignault et al., 2001; Muchembled et al., 2006). Plant cell derived T-6-P acts as a molecular switch that negatively regulates the activity of the sucrose non-fermenting related protein kinase 1 (SnRK1). SnRK1 is a master regulator that controls sugar metabolism during biotic stresses (Hulsmans et al., 2016). KIN 10 and KIN 11 the *Arabidopsis* analogs of SnRK1, which have been seen to be functional under both types of stress, establish a link between sugar metabolism and the metabolic disruptions seen under pathogen attack. Rice cultivars sensitive to *Magnaporthe grisea*, for instance, were seen to have fewer metabolites involved in sugar metabolism, compared to the resistant varieties (Jones et al., 2011). It was later shown by Baena-Gonzalez (2010) that SnRK1 is the primary modulator repressing the energy intensive synthetic pathways and activating catabolic pathways under stressed conditions, to restore cellular homeostasis . Interestingly, the effect of SnRK1 activation, under abiotic stresses could be reversed by the exogenous application of sucrose. Inhibition was also seen upon the external application of glucose and glucose-6-phosphate (G-6-P) (Zhang et al., 2009; Nunes et al., 2013). This repression was attributed to the fact that drops in levels of sucrose, glucose, G-6-P and T-6-P were acting as starvation signals which in turn induced SnRK1. In *Arabidopsis* seedlings, the increased T-6-P levels act as a “feast signal” suppressing SnRK1 activity (Morkunas and Ratajczak, 2014).

SnRK1 has also been shown to drive the expression of stress inducible genes in plants aided by the heterodimerization of S-group and C-group bZIP transcription factors (for basic region/Leu zipper motifs) leading to protection against pathogens via PCD. The response of the transcription factors to sugar levels is, however, variable as seen for *Arabidopsis At*bZIP11, which is sugar inducible, while *At*bZIP1, *At*bZIP2, and *At*bZIP53 are sugar repressible. As new insights are drawn regarding the role of sugars in modulating the metabolic state and defense responses under pathogen attack, gaps in the understanding of signaling pathways continue to emerge. With respect to sucrose, glucose, G-6-P, and T-6-P there is sufficient evidence to suggest that they function as signals reflecting the general metabolic health of the plants and also behave as systemic defense modulators in certain cases. However, various questions remain unanswered regarding the site and mechanism of action for these glycans, as well as their holistic roles in plant defense.

### Galactinol, Raffinose, and Related Oligosaccharides

**FIGURE 4:**
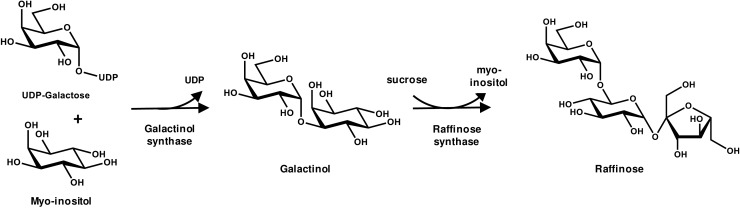
The biochemical pathway of Raffinose Oligosaccharide synthesis.

Galactinol in plants is present as a precursor for the synthesis of the raffinose family oligosaccharides (**Figure 4**), which serve as an osmoprotectant of plants, as a transporter of sugar in phloem sap, and as storage sugars (Sengupta et al., 2015). Soybean plants under drought stress conditions, when pretreated with H_2_O_2_, were seen to have increased levels of galactinol which is known to function as an ROS scavenger (Ishibashi et al., 2011; Savvides et al., 2016). A similar phenomenon was also observed in *Arabidopsis* where oxidative stress induced the expression of high levels of galactinol synthase (*GolS*) (Nishizawa et al., 2008; Petrov et al., 2015). This enzyme catalyzes the first step toward the biosynthesis of galactinol which was first detected in a crude extract of maturing pea seeds (Zhou et al., 2017). The induction of the *GolS* gene leading to the biosynthesis of galactinol has mostly been studied in relation to osmotic stresses across various plants such as *Brassica napus* L., *Coffea arabica* L., *Salvia miltiorrhiza*, grapevine, *Medicago* *falcata*, chestnut, and *Cicer arietinum* L. The first evidence of galactinol being involved in inducing systemic resistance during biotic stress was observed during the interaction between *Pseudomonas chlororaphis* and cucumber or tobacco plants. Galactinol enhanced the accumulation of defense-related genes *PR1a*, *PR1b*, and *NtACS1* (*Nicotiana tabacum* 1-aminocyclopropane-1-carboxylic acid synthase 1), which in turn enhanced resistance against *Botrytis cinerea* and *Erwinia carotovora* in pathogen challenged cucumber and tobacco plants (Kim et al., 2008). Preliminary investigations on *Camellia sinensis GolS* gene (*CSGolS*) have shown that *CsGolS1* regulation was mainly related to abiotic stresses such as water deficiency, low temperature, and ABA treatment. On the other hand, significant regulation of *CsGolS2* and *CsGolS3* was seen in relation to biotic stresses such as *E. oblique* (moth) attack, SA, and MeJA treatment (Zhou et al., 2017). Although the *GolS* gene has been extensively studied under abiotic stress, the regulation of the galactinol levels by this gene during biotic stress (pest attack and microbial invasion) needs to be further investigated to understand the underlying mechanisms. Furthermore, the roles played by galactinol derivatives in response to stresses including pathogen attack remain to be elucidated (Sengupta et al., 2015).

### Polyols

**FIGURE 5:**
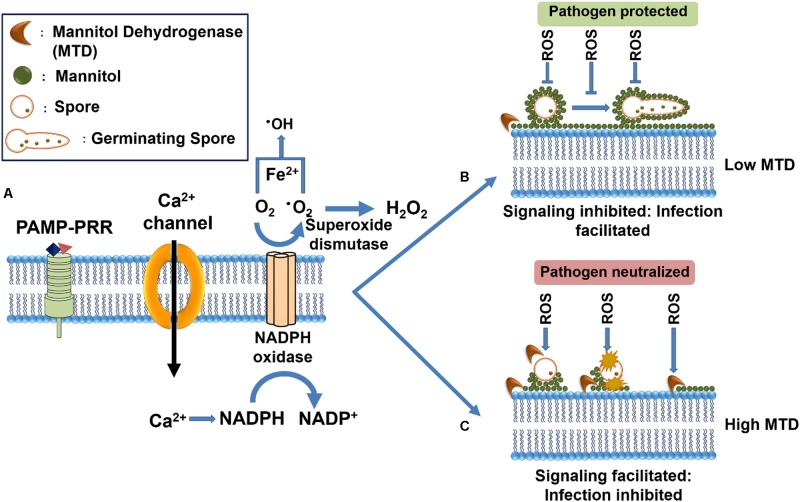
Illustration of the influence of Mannitol and Mannitol dehydrogenase in mediating plant defense responses. **(A)** Recognition of fungal PAMPs in the host apoplastic space triggers PTI mediated oxidative burst which is characterized by the activation of a membrane-bound NADPH oxidase, leading to the generation of reactive oxygen species (ROS) superoxide (∙O_2-_), which is subsequently converted into hydrogen peroxide (H_2_O_2_) and hydroxyl radical (∙OH). **(B)** The filamentous pathogens secrete mannitol in response to mannitol dehydrogenase oxidative burst to mitigate host mediated oxidative burst through the formation of a protective shield around the fungal structures. **(C)** Several plants have been seen to secrete a mannitol catabolic enzyme, mannitol dehydrogenase (MTD) that can metabolize the fungal mannitol, thereby restoring the ROS-mediated responses and including antimicrobial effects (Adapted from Patel and Williamson, 2016 and reproduced with permission from Elsevier).

Polyols, often referred to as sugar alcohols, are the reduced form of aldose and ketose sugars (Noiraud et al., 2001). Mannitol is a soluble polyol which is structurally related to the aldohexose mannose. It is widely present in bacteria, algae, fungi, and more than 100 species of higher plants including many crops such as celery, olive, and carrot (Patel and Williamson, 2016). Mannitol is an osmoprotectant, contributing to salt tolerance in plants and it has been demonstrated to be an *in vitro* quencher of reactive oxygen species that limits cell damage (Jennings et al., 2002). Interestingly, mannitol is also a common metabolite in most filamentous plant pathogens including ascomycetes, basidiomycetes, deuteromycetes, and zygomycetes (Jennings, 1985; Solomon et al., 2007). The fungal pathogens *Alternaria alternata*, *A. brassicicola*, and *Cladosporium fulvum* are reported to secrete mannitol when they encounter plant tissues or extracts (Williamson et al., 2013). Mannitol accumulation was first documented in the apoplast of tomato leaves upon infection with a virulent strain of *C. fulvum*, but was absent in leaves infected with the avirulent stain (Joosten et al., 1990). The role of mannitol as a pathogenicity factor was further consolidated by reports stating greatly reduced virulence of *A. alternate* mannitol synthesis knockout mutants (Δ*mtdh*/Δ*m1pdh*) (Vélëz et al., 2007). In light of such reports, mannitol is speculated to be involved in quenching ROS-mediated plant defenses by virtue of its antioxidant properties (Calmes et al., 2013). The current opinion in this regard suggests that mannitol secretion by filamentous pathogens serves as a self-defense strategy against plant defense responses and may also be a means of obstructing the plant defense signaling pathways by blocking plant mediated ROS signals (Juchaux-Cachau et al., 2007). This aspect has been recently reviewed by Patel and Williamson (2016) to present a mechanistic hypothesis (**Figure 5**) regarding the interplay between mannitol and mannitol dehydrogenase during pathogenesis of the mannitol-secreting pathogens and how it may be balanced between detection of pathogens by plants and evasion of defense response by the pathogen.

## Oligosaccharides

Most oligosaccharides implicated in plant–pathogen interactions are generated by the enzymatic degradation of polysaccharides from the structural constituents of fungal cell wall or pathogen virulence factors (Zhang S. et al., 2015). The activity of these oligosaccharides is highly dependent on their degree of polymerization (DP) (Trouvelot et al., 2014).

### β-1,3-/ β-1,6-glucans

β-1,3-/ β-1,6-Glucans based oligosaccharides have been extensively explored in view of their involvement in plant–pathogen interactions for several decades (Fesel and Zuccaro, 2016). β-glucans were first isolated from crude or enriched fractions of *Phytophthora sojae* mycelial cell wall hydrolysate, which mediated plant defense by the induction of PAL activity in plants (Ayers et al., 1976; Ebel et al., 1976). In tobacco and *Arabidopsis*, sulfated β-1,3-glucans were able to induce a SA mediated defense reactions conferring resistance to tobacco mosaic viruses (Menard et al., 2004). Laminarin, a β-1,3-glucan, derived from the brown algae *Laminaria digitata* was seen to elicit a variety of defense reactions in tobacco plants. Treatment of tobacco with laminarin as an elicitor led to the enhanced activity of PAL, caffeic acid, *O*-methyl transferase, and lipoxygenase, accumulation of SA, and transcriptional activation of PR proteins. These events resulted in activation of induced resistance against the soft rot pathogen *E. carotovora* (Klarzynski et al., 2000). Using laminarin elicitors on grapevine induced early defense responses including calcium influx, oxidative burst, extracellular alkalization of the culture medium, and activation of mitogen-activated protein kinases (MAPK) (Aziz et al., 2003). Laminarin in later stages of host defense induced the expression of defense genes associated with the octadecanoid and phenylpropanoid pathways leading to a significant protection of grapevine leaves against *B. cinerea* and *Phomopsis viticola*. However, HR mediated cell death was totally absent in these plants (Aziz et al., 2003). In most cases β-1,3-glucans and laminarin have DPs between 10 and 16 which are often referred to as optimal for induction of plant defense responses (Navazio et al., 2002; Galletti et al., 2008; Vorholter et al., 2012). However, Fu et al. (2011) have demonstrated using tobacco cell suspensions that β-1,3-glucans having DPs as low as 2–10, provide a higher protection against tobacco mosaic virus compared to those with higher DP (25–40) (Fu et al., 2011). Similarly, in *Oryza sativa* cell suspensions β-1,3-glucan oligomers with a DP ≥4 were shown to stimulate chitinase activity while those with DP 6 acted as inducers of PAL activity (Inui et al., 1997).

Plants have been seen to respond differently to structurally distinct forms of β-glucans, as seen in soybean and rice which are able to recognize only branched β-glucans (Cheong and Hahn, 1991; Yamaguchi et al., 2013) whereas tobacco recognizes the linear β-1,3-glucans. With respect to the response to chemically modified glucans as elicitors, acetylated oligoglucuronans having DP ≥14 could induce the transient production of H_2_O_2_ and defense gene expression (PAL, Chitinase and Polygalacturonase inhibiting protein). This strategy was effective in reduction of *B. cinerea* infection of grapevine leaves upon treatment with these acetylated oligoglucuronans (Caillot et al., 2012). In the case of natural laminarin, chemically sulfated analogs with DP >5 were seen to be effective in stimulating the SA signaling pathway leading to protection against pathogens in tobacco and *Arabidopsis* plants (Menard et al., 2004). A hepta-β-glucan isolated from culture medium during germination of *Phytophthora megasperma* f. sp. *glycinea* was found to elicit the synthesis of isoflavonoid phytoalexins in soybean cotyledons (Sharp et al., 1984a,b). This interaction was seen to stimulate the induction of localized HR mediated resistance against *P. megasperma* f. sp. *glycinea* in several other legumes namely soybean, alfalfa (*Medicago sativa*), bean (*Vicia faba*), lupin (*Lupinus albus*), pea, *Medicago truncatula*, and *Lotus japonicus* (Cosio et al., 1996; Cote et al., 2000).

The recognition of fungal β-glucans by animal cells is known to induce the production of inflammatory chemokines/cytokines such as TNFα, IL-1b, IL-10, IL-6, IL-23, CCL2, CCL3, etc., when perceived by the homo-dimerization and phosphorylation of Dectin-1 (Brown and Gordon, 2003; Drummond and Brown, 2011). Cell signaling in case of Dectin-1 can proceed through both SYK-dependent (Spleen Tyrosine Kinase) and independent pathways (RAF1-dependent), bringing about a horde of effects ranging from internalization of pathogen for antigen-presentation to cytokine/caspase activation. Dectin-1 consists of an extracellular C-type lectin domain that is connected to the plasma membrane by a stalk region which protrudes in to the extracellular space. The cytoplasmic C-terminus contains an immunoreceptor tyrosine-based activation motif (ITAM). Dectin-1 is primarily localized on the surface of macrophages and to a lesser extent on dendritic cells that binds to β-1,3-glucans and mixed β-1,3/1,6-glucans like laminarin, zymosan, and complete yeast cells. The intracellular ITAM motif is phosphorylated by SRC, a tyrosine-protein kinase, upon recognition of β-1,3-glucans which activates the downstream Syk signaling cascade leading to immunogenic responses (Levitz, 2010). However, the phosphorylation of ITAM is preceded by the dimerization of two Dectin-1 receptors, following β-1,3-glucan binding (Brown et al., 2007). The interaction between β-glucan and Dectin-1 is mediated through the Trp221 and His223 amino acids on the C-type lectin domain of Dectin-1 (Adachi et al., 2004) which shares a 30% homology with *A. thaliana* and soybean, C-type lectin (Greeff et al., 2012; Singh and Zimmerli, 2013). However, the structure of the β-glucan binding groove in the corresponding plant C-type lectin domain is not known and it is speculated that the binding of a more complex β-glucan could be accommodated by a different set of amino acids in the plant Dectin-1 orthologs (Brown et al., 2007). Dectin-2 and Dectin-3 hetero-dimers were reported to recognize α-mannans as PAMPS, during the *Candida albicans* infection of mice. The recognition of α-mannans by Dectins 2 and 3 is mediated by the ITAM-containing cytoplasmic adaptor protein FcRγ, as they themselves are devoid of the ITAM motifs on the cytoplasmic end. The activation and phosphorylation of the Dectin 2 and 3 was reported to induce the production of NF-κB and pro-inflammatory cytokines such as TNF-α, IL10, IL12, and IL-6 (Zhu et al., 2013). However, no plant analogs for Dectin 2/3 have been reported so far.

**FIGURE 6:**
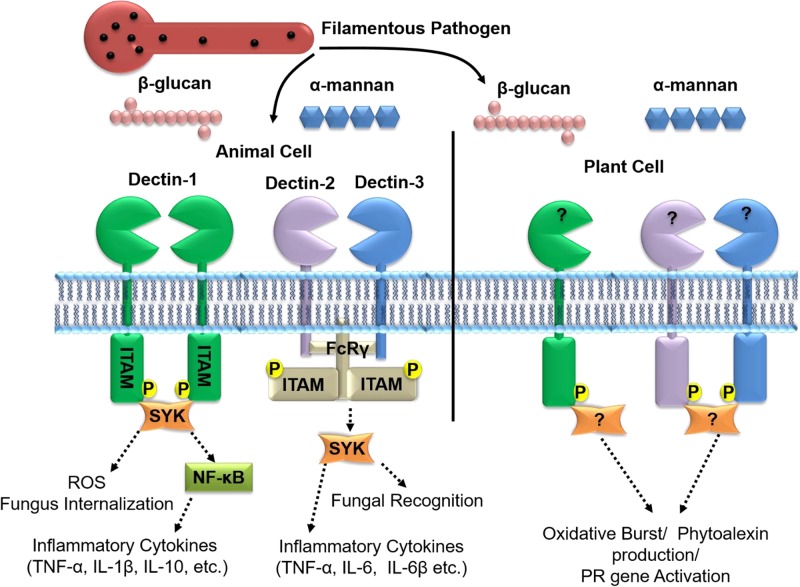
β-glucan and α-mannan mediated signaling in animal cells alongside the putative system in plants. In animal cells, the recognition of β-glucan is mediated through Dectin-1 homo-dimerization followed by the phosphorylation of its cytoplasmic Immuno-receptor Tyrosine-Based Activation (ITAM) domain and downstream cell signaling. Activation of Dectin-1 signaling can lead to stimulation of ROS pathways, internalization of pathogen by cells and activation of chemokine/cytokines/caspases. α mannans on the other hand are recognized by the hetero-dimerization of Dectin-2 and Dectin-3 followed by the phosphorylation of the FcRγ-ITAM motifs and downstream signaling that triggers the production of pro-inflammatory cytokines (TNF-α, IL10, IL12, and IL-6, etc.). Although little is known about β-glucan perception in plants, studies suggest β-glucan treatment leads to the defense responses that include generation of H_2_O_2_, production of phytoalexins and activation of PR-genes. It is not clearly known if homologs of Dectin-1/2/3 are functional in the recognition and defense signaling in responses to the filamentous pathogens of plants. The dotted lines represent the signaling pathways involved.

Although various findings have shown a prominent role of β-glucan in priming plant defense, the putative sequence and domain structure for the plant β-glucan receptor is not known. One of the reasons could be that such studies have mostly been restricted to the Col-0 ecotype of *A. thaliana*, which is not known to elicit a strong immune response against β-glucan treatment (Fesel and Zuccaro, 2016). Furthermore, the work done on β-glucan mediated immunity during fungus–animal interactions appears to provide leads toward the better understanding of β-glucan perception in plants, wherein plant Dectin-1 homologs are believed to harbor a mammalian SYK-like domain, instead of the ITAM domain (Fesel and Zuccaro, 2016). Based on the information available, a schematic depicting the β-glucan and α-mannan mediated immunity in animals and the hypothetical model of its plant counterpart is depicted in **Figure 6**. A genome wide survey of the available *Arabidopsis* ecotype resources or investigation of the β-glucan and α-mannan perception in other model plants could possibly unearth the missing links to complete the picture.

### Cellodextrins

Cellodextrins are a group of oligosaccharides that consist of a linear β-1,4- linked glucose backbone, which are the end-products of cellulose degradation in plants. It has been reported that cellodextrin treatment of plant cells leads to the activation of PAL genes, which enhances synthesis of lignin, SA, and phytoalexin as an innate immune response. They in turn activate acidic (Chit3, Chit4c) and basic (Chit1b) chitinases and β-1,3-glucanase which are responsible for hydrolysis of chitin and β-1,3-glucans in fungal cell walls (Farmer et al., 1991; De Lorenzo and Ferrari, 2002). Exogenous application of cellodextrin as an elicitor was also seen to activate protection against *B. cinerea* by triggering induction of oxidative burst, defense-related genes, elevation of cytosolic Ca^2+^, and stimulation of chitinase and β -1,3-glucanase activity (Aziz et al., 2007). In grapevine, the generation of H_2_O_2_ and subsequent oxidative burst was highest in response to heptasaccharide cellodextrins (Aziz et al., 2007). Also, in *Arabidopsis*, treatment with cellobiose induced the innate immune responses such as increased cytosolic calcium and activation of MAPK pathway in response to *Pseudomonas syringae* infection (De Azevedo Souza et al., 2017). However, the fundamentals of cellodextrin signaling still remain unclear and the correlation between the degree of polymerization and intensity of defense response needs further investigation.

### Chitin and Chitin Derivatives

Chitin, a polymer of *N*-acetylglucosamine, is a major constituent of fungal cell walls and is the most well characterized filamentous PAMP (Shibuya and Minami, 2001). It has been seen to stimulate phytoalexins and trigger the MAPK based defense signaling in response to *Magnaporthe oryzae* challenge of rice, thereby providing a systemic resistance (Yamada et al., 1993; Yamaguchi et al., 2013). The first PRR for chitin, Chitin Elicitor Binding Protein (*Os*CEBiP), was identified as a plasma membrane localized receptor in rice cells, which was responsible for defense signal transduction (Miya et al., 2007; Kishimoto et al., 2010). In barley, the basal resistance against *M. oryzae* was seen to develop upon the recognition of chitin by the *Os*CEBiP homolog, *Hv*CEBiP (Tanaka et al., 2010). The chitin PRR in *Arabidopsis*, *At*CERK1 (also known as RLK1/LYK1) contains additional extracellular carbohydrate binding lysine motifs (LysMs), as well as an intracellular kinase domain essential for chitin based defense signaling (Egusa et al., 2015). Recent discoveries on *Arabidopsis* PRR have shown that *At*CERK receptor also mediates recognition of 1,3-β-D-(Glc)_6_, a hexasaccharide from the necrotrophic fungus *Plectosphaerella cucumerina* which induces innate defense responses such as increased levels of cytosolic Ca^2+^, activation of MAPKs cascades and elevated expression of PR proteins (Melida et al., 2018). Chitin oligosaccharides when used as elicitors were reported to induce the expression of pathogenesis-related protein, PR-10 and generation of ROS in both suspension-cultured rice cells and *Arabidopsis* seedlings (Akimoto-Tomiyama et al., 2003). In a recent study, the interaction between *Phytophthora palmivora* and *L. japonicus* led to the rapid transcript accumulation of LYS12, a LysM receptor protein of the NFR5-type, which could control the progression of disease by regulating defenses genes like POX, Germin-like protein, and Chitinase. Further, LYS12 mutants responded to treatments with glycan elicitors such as chitin oligomers and β-1,3- and β-1,6-glucan. It is speculated that there could be other functional components in the perception and signaling of these elicitors as LYS12 lacks the ATP binding loop and the typical Asp-Phe-Gly (DFG) motif in subdomain VII, essential for signaling. Also, several LysM receptors have been found to recognize and distinguish pathogenic/mutualistic carbohydrate signatures emerging from GlcNAc derived and microbial exopolysaccharide sources. It is therefore plausible that LYS12 is involved in monitoring carbohydrate PAMPs produced by *P. palmivora* as well as the damage associated signatures emerging from the host plant, thereby regulating disease progression (Fuechtbauer et al., 2018).

Chitosan, β-1,4-linked glucosamine is a deacetylated derivative of the chitin that forms a major component of many fungal cell walls, including those of fungal pathogens. Chitosan or its fragments have been shown to induce defense responses in dicotyledonous plant species like soybean and parsley mediated by the synthesis of callose, a β-1,3-glucan polymer (Kohle et al., 1985; Conrath et al., 1989). In melon plants, chitosan oligomers were shown to stimulate chitinase activity, while in wheat, treatment with chitosan oligosaccharides induced lignin deposition and increased phenolic acids level, in leaves (Burkhanova et al., 2007).

Chitin oligosaccharide derivatives bearing lipid modifications are known as lipochitooligosaccharides and act as microbial signals to initiate symbiotic association of arbuscular mycorrhizal symbiosis (Myc factors) and root-nodule symbiosis (Nod factors) (Zipfel and Oldroyd, 2017). On the other hand, chitin oligosaccharide tetramers and heptamers have been reported to induce oscillations of Ca^2+^ levels in the cell nucleus during symbiotic association in various legumes pea, *M. truncatula* and *L. japonicus* and rice (Sun et al., 2015). Thus chitin and its oligosaccharide derivatives acting either as defense elicitors or symbiosis activating signals may begin to answer how plants distinguish between friendly and pathogenic interactions in the apoplastic space. However, the role of the glycans in the interplay between the defense and symbiotic signaling networks and how plant signaling processes discern and deliver specific outcomes, remains to be unraveled.

### Alginate and Fucans

Alginate oligomers are formed by depolymerization of alginates obtained from sea weed that are made up of alternating blocks of L-glucuronic and D-mannuronic residues (Gonzalez et al., 2013). Alginate oligomers are gaining increasing attention as new elicitor materials for inducing plant defense machinery by stimulating the accumulation of phytoalexin and inducing PAL activity in soybean cotyledons (An et al., 2009). Furthermore, alginate oligosaccharides rich in polymannuronate are reported to activate defense responses in *O. sativa* against *M. oryzae* by inducing increased level of PAL, phytoalexin production, and POX activity (Zhang S. et al., 2015).

Sulfated fucan oligosaccharides made up of mono- and di-sulfated fucose units alternatively bound by α-1, 4- and α-1,3- glycosidic linkages have been shown to be strong inducers of PAL, in tobacco cell suspension cultures (Kombrink and Somssich, 1995; Klarzynski et al., 2003). Inoculation of tobacco leaves with these fucan oligosaccharides resulted in the accumulation of several PR proteins and the development of jasmonate mediated systemic resistance toward TMV (Klarzynski et al., 2003).

## Glycoprotein and Lectins

Alongside secreted protein effectors that destroy plant cell integrity filamentous pathogens are known to secrete small proteinaceous molecules that induce necrosis, shrinkage of cytoplasm, silencing of defense genes, electrolyte leakage, and generation of ROS in their hosts. These secreted molecules not only contribute to invasion and absorption of nutrients, but also assist in establishing necrotrophic/hemibiotrophic lifestyles by inducing HR-mediated cell death (Gonzalez et al., 2017). The first report of glycoproteins being involved as an elicitor that induced the defense related phytoalexin production was identified in *P. megasperma* and parsley interactions (Nurnberger et al., 1994). Much later the glycoprotein, *BcGs*1 produced from the culture filtrate of the necrotrophic fungus *B. cinerea* was seen to act as a necrosis-inducing elicitor that activated the typical HR as well as components of the SA, JA, and ET defense pathways, in tomato. *BcGs*1 treatment of tomato plants was found to induce the SA- dependent defense marker *PR-1a*, Prosystemin, a known elicitor of JA defense signaling and the tomato protein kinase 1 (TPK1b), which is an ET-mediated shared defense signal for protection against necrotrophic fungi and herbivorous insects. (Zhang Y. et al., 2015). However, the interaction between the SA, JA, and ET defense pathways, for rendering defense remains to be investigated. Earlier studies on the *B. cinerea* protein secretome have also shown the presence of an abundantly secreted glycoprotein, *Bc*IEB1, with unknown function (Espino et al., 2010). Work on the structural analysis of the *Bc*IEB1 glycoprotein has shown two serine/threonine-rich residues glycosylated with α-1,2-/ α-1,3-linked mannose (Gonzalez et al., 2012, 2014). Recently, the elicitor function of *Bc*IEB1 glycoprotein was discovered by Frias et al. (2016), wherein *Bc*IEB1 expressed in yeast when assayed as a purified elicitor on tobacco, tomato, onion, and *Arabidopsis*, was able to induce defense responses against *B. cinerea.* The elicitor activity of the *Bc*IEB1 protein induced ROS burst, electrolyte leakage, seedling growth inhibition, cytoplasm shrinkage and cell autofluorescence (Frias et al., 2016). Further studies on the *Bc*IEB1 elicitor by Gonzalez et al. (2017) led to the discovery of the tobacco plant osmotin, a stress response protein, a member of the PR5 family, as the PPR of *Bc*IEB1 glycoprotein. In a separate study, osmotin also was found to accumulate in plants as a response to invasion of fungal pathogens, leading to activation of PCD in fungi, lysis of fungal membranes and promoting the hyperaccumulation of osmolyte proline, which quenches ROS. Hence, the roles and diversity of effectors and their corresponding plant receptors extend well beyond the conventional effectors of a proteinaceous nature to involve many diverse molecules and pathways.

Glycan-binding lectin proteins are reported to regulate many of the defense signaling pathways during host–microbe interactions and immune responses (Silipo et al., 2010). Lectins mediate the recognition of plant pathogens upon perception of characteristic epitopes or damage-associated patterns, using protein–protein interactions as well as protein-glycan interaction (Lannoo et al., 2014). Lectin domain containing receptor-like kinases (LecRLKs) involved in pathogen recognition including LecRK-I.9 from *Arabidopsis* and Pi-d2 from rice (*O. sativa*) are reported to act against the oomycete pathogen *Phytophthora brassicae* and the ascomycete *M. oryzae*, respectively (Weidenbach et al., 2016). In LecRK, the extracellular lectin domain of LecRK is composed of a conserved hydrophobic groove that helps in the recognition of glycans (Andre et al., 2005; Wang and Bouwmeester, 2017). LecRK-I.9 domain in *Arabidopsis* interacts with oligosaccharides via two tripeptide Arg-Gly-Asp (RGD) in the lectin domain (Gouget et al., 2006). Also, extracellular ATP released by plants upon pathogen invasion acts as a ligand perceived by LecRk-I.9 domain (Choi et al., 2014). However, insights into the mechanism regulating both of these processes and how pathogens adapt to LecRK-mediated defense remains to be seen.

## Glycolipids

Lipopolysaccharide (LPS) is a vital structural component of the outer layer of Gram negative bacteria with three distinct functional domains which include: the lipophilic lipid A (LA) the di-glucosamine moiety which carries 4–7 fatty acids, an oligosaccharide core region, and the *O*-antigen region consisting of a variable number of oligosaccharide repeats (Alexander and Rietschel, 2001). The oligosaccharides in LPS act as a signaling moiety during induced immunity while the LA domain is recognized, even at picomolar concentrations, as a PAMP through different extra- and intra-cellular LPS sensors (Whitfield and Trent, 2014). The LPS sensing receptor in plants is RLK LORE (Lipooligosaccharide-specific Reduced Elicitation), which belongs to the plant-specific class of bulb-type lectin S-domain-1 kinases (SD-RLKs). RLK LORE can sense *Pseudomonas* LPS as PAMP in *Arabidopsis* and other crucifers triggering typical PTI responses (Ranf et al., 2015). The significance of RLK LORE in LPS perception was demonstrated by restoration of LPS sensitivity in LPS-insensitive tobacco plants through the expression of RLK LORE.

**FIGURE 7:**
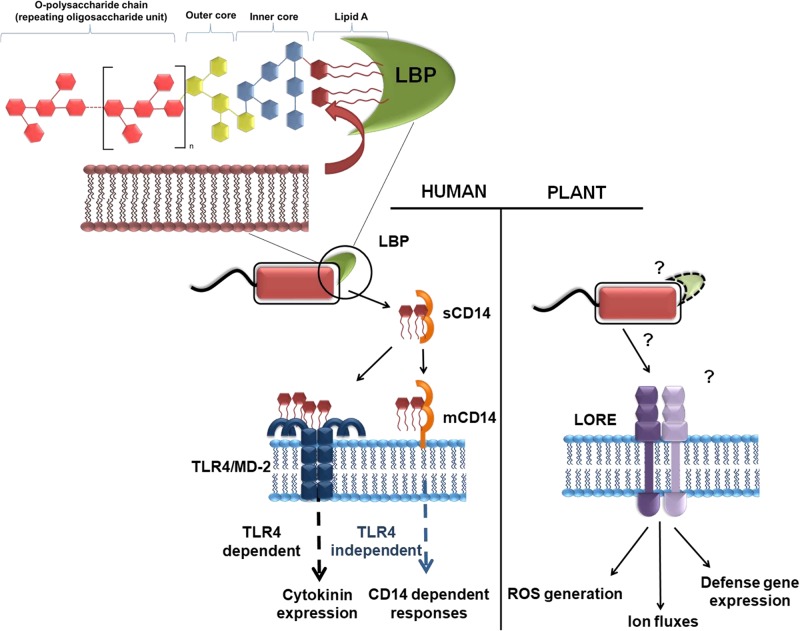
Mechanism of LPS receptor in human and plant systems. In humans, the lipid A (LA) domain of LPS is disaggregated from the bacterial membrane by the serum protein LBP and transferred to CD14, which occurs as a soluble (sCD14) and membrane-linked (mCD14) version. CD14 can transfer LPS to the membrane-resident TLR4/MD-2 receptor complex. Depending on the cellular localization (at the plasma membrane or in endosomes upon CD14-dependent endocytosis), TLR4/MD-2/LPS complexes activate production of either interferons or cytokines through distinct signaling adapters (TIRAP/MyD88 or TRIF/TRAM). In some cell types CD14 can directly trigger immune responses. In plants, the bulb-type lectin S-domain-1 RLK LORE (Lipooligosaccharide-specific Reduced Elicitation) was identified as the first LPS receptor component in plants and mediates sensitive perception of LA domain of Pseudomonas. However, it is yet unknown whether LA directly binds directly to LORE or to an LPS-binding co-receptor to activate the receptor complex and downstream signaling [Adapted from Ranf, 2016 and reproduced with permission under Creative Commons Attribution (CC BY) license].

LPS mediated defense signaling is well characterized in the mammalian system where it binds to the LPS receptor, TLR4/MD-2 (toll-like receptor 4/myeloid differentiation factor-2), to trigger activation of phagocytosis, production of pro-inflammatory cytokines, and interferons and antimicrobial peptides (**Figure 7**; Beutler et al., 2001; Tan and Kagan, 2014). The inflammatory cascade can be activated directly by the recognition of the bacterial LPS by the mammalian glycoprotein CD14, in a TLR independent fashion. Alternatively, CD14 mediates the transfer of the bacterial LA by the mammalian serum protein LPS-binding protein (LBP), in TLR dependent fashion. Taking inference from mammalians LPS signaling, it is currently believed that LORE undergoes dimerization during LPS perception. However, it is still not clear if any other plant defense modulators facilitate the transfer of the LA or facilitate LPS recognition and signaling in a LORE-independent manner.

Galactolipids, which are primarily composed of monogalactosyldiacylglycerol (MGDG) and digalactosyldiacylglycerol (DGDG), are important plant glycerolipids, known for their role in mediating in systemic acquired resistance (SAR) based plant defenses. It was seen that *Arabidopsis mgd1* mutant which is deficient in MGDG synthesis and the *dgd1* mutant, deficient in DGDG synthesis could not mount a SAR mediated defense response, when challenged with a secondary infection of the virulent pathogen *P. syringae* pv. *maculicola* after they were primed with the avirulent pathogen *P. syringae* pv. Tomato (Chaturvedi et al., 2008; Gao et al., 2014). This provides a strong evidence regarding the role of MGDG and DGDG in establishing SAR during pathogen attack; however, the complete pathway remains to be elucidated.

Phosphoinositol sphingolipids and glucosylceramides (GlcCer) are another group of glycosylated lipids found in fungi that feature a characteristic C-9 methyl group present on the long chain fatty acid base (Warnecke and Heinz, 2003). These fungal GlcCer behave as elicitors that induce plant defense responses like phytoalexin production and PR protein synthesis, when sprayed on to rice (Umemura et al., 2000).

Natural rhamnolipids produced by *Pseudomonas aeruginosa* have been shown to induce defense responses in grapevines, tobacco, and *Arabidopsis* cells via oxidative burst, ROS production, and inducing systemic acquired resistance (Varnier et al., 2009; Sanchez et al., 2012). In this context, natural rhamnose based glycolipids were synthesized enzymatically to use as elicitors of defense response and were found to induce extracellular ROS production when tested on tobacco cells (Nasir et al., 2017).

## Conclusion

Our current understanding of the roles that carbohydrates play in PAMP based defense priming in plants remains fragmented. Although the potential role of glycans as elicitors of plant defense signaling cascades has been identified as an important aspect, there remains a huge information gap when compared to what we currently understand about proteinaceous receptors of these glycan elicitors. The likely reason for this is the complexity of carbohydrate structures and the fact that the techniques used to analyze glycans lag behind those for protein and gene analysis. Improvements in oligosaccharide extraction, purification and identification techniques are beginning to enable studies using more specific glycan species rather than crude extracts containing polydisperse carbohydrate species. The understanding of the roles played by carbohydrates in plant–pathogen interactions falls behind knowledge in human–pathogen interactions, which has attracted attention due to the implications in medicine development and disease treatment. It is only recently that we have begun to realize the importance carbohydrates have in plant protection strategies and appreciate the potential that carbohydrates have as specific intervention technologies.

In their natural habitat, plants are exposed to a wide variety of symbiotic and pathogenic microflora at any given time, which paves the way for a novel and extensive area of research to investigate the controlled and diversified signaling cascades generated within plants in response to simultaneous attack from different microbes. Plant genomes encode a large number of RLKs, RLPs, and lectins; biochemical and structural studies on these receptors will lead to better knowledge of their workings and allow identification of the most suitable oligosaccharide candidates.

Coming to the concept of sustainable agriculture, there is potential to use oligosaccharides for crop protection, to induce pathogen resistance and prime plants for microbial attack. The use of biodegradable, environmentally friendly glycans and glycan mimics will help to replace the conventionally used chemicals with safer alternatives. This will enable more sustainable agriculture and help avoid major crop losses, allowing better food security, given mankind’s growing demand for food quality and quantity.

## Author Contributions

CC, EK, and MR conceived the idea and prepared the draft of the manuscript. RF supervised the development of topics for discussion and edited the draft manuscript.

## Conflict of Interest Statement

The authors declare that the research was conducted in the absence of any commercial or financial relationships that could be construed as a potential conflict of interest.
